# Implicit and explicit attitudes toward gay men and lesbian women among heterosexual undergraduate and graduate psychology and nursing students

**DOI:** 10.3389/fpsyg.2022.921313

**Published:** 2022-07-28

**Authors:** Oz Hamtzani, Yaniv Mama, Ayala Blau, Talma Kushnir

**Affiliations:** ^1^Department of Psychology, Ariel University, Ariel, Israel; ^2^Department of Nursing, Ariel University, Ariel, Israel; ^3^Adelson School of Medicine, Ariel University, Ariel, Israel

**Keywords:** attitudes, sexual orientation, psychology, nursing, students

## Abstract

**Objectives:**

To examine implicit and explicit attitudes toward gay men and lesbian women among heterosexual undergraduate and graduate psychology and nursing students.

**Methods:**

Implicit attitudes were measured *via* the Implicit Association Test and explicit attitudes *via* the Attitudes Toward Lesbian Women and Gay questionnaire.

**Main results:**

All groups held negative implicit attitudes toward gay men and lesbian women. Among undergraduates, nursing students reported holding more negative explicit attitudes toward gay men and lesbian women than psychology students.

**Conclusion:**

The curricula in both nursing and psychology studies need to address the medical and paramedical needs and issues of sexual minorities.

## Introduction

In many countries, there are laws and ethical codes currently in place to ensure proper and equal medical care for all patients (American Nurses Association, 2015; World Health Organization [WHO], 2015; American Psychological Association [APA], 2017). Although the implementation of such laws is enforced, many patients complain of receiving unequal medical treatment from various health care providers. Studies found prejudice, bias, and negative stereotypes among medical staff members toward disadvantaged minority population groups such as drug and alcohol addicts, overweight patients, patients with mental health disorders, patients of different cultural origins, and sexual minority patients (Sabin et al., 2009; Budd et al., 2011; Haider et al., 2011; Sabin and Greenwald, 2012; Van Boekel et al., 2013).

Sexual minority patients are at increased risk for mental and physical health issues (Garnero, 2010; Mor et al., 2015). Compared with heterosexuals, sexual minority patients suffer at higher rates from obesity, lack of physical activity, eating disorders, mental health disorders, dissatisfaction with appearance, unhealthy use of alcohol and drugs, and take more risks in sexual activities. Yet, many sexual minority patients hesitate to turn to medical and paramedical services. Among the main reasons are lower levels of trust in the medical system due to awareness of prejudice against them in society (Williams and Mohammed, 2013; Mor et al., 2015; Sabin et al., 2015; Casey et al., 2019). Ayhan et al. (2020) performed a systematic review of the literature regarding discrimination against sexual minority patients in a health care setting. The results indicated that sexual minority patients experienced discriminative behaviors such as stigma, denial, refusal of health care, and abuse.

Prejudice is defined as an unjustified negative attitude based on a person’s group membership (Institute of Medicine, 2002). Studies found negative attitudes toward gay men and lesbian women among a variety of health care providers such as nurses (Dorsen, 2012; Strong and Folse, 2015; Unlu et al., 2016; Tzur-Peled et al., 2019), mental health professionals (Jones, 2000; Bowers et al., 2015; Tan et al., 2017), physicians (Jabson et al., 2016) and others health care providers (Nathan et al., 2019). In these studies, attitudes were usually assessed *via* direct measures such as self-reports and surveys.

Attitude assessment using self-reporting methods may not reveal the true nature of a person’s attitudes. This may be one of the explanations why researchers in the field claim that in recent years there has been a marked decline in expressions of stereotypical beliefs and discrimination (Dovidio et al., 2016), while paradoxically, as shown earlier, sexual minority patients claim that they are being discriminated against by health-care providers.

Following such inconsistencies, in recent years there has been a turnaround in the research literature in examining the influence of attitudes on discrimination and stereotyping behaviors. Prominent theoreticians have suggested that in order to investigate a person’s true attitude, one must use an indirect measure since subjects are not always aware of the real nature of their attitudes. In addition, responses to direct measures may be biased by social desirability so that they do not reflect true attitudes (Eagly and Chaiken, 2007; Ajzen et al., 2018). Consequently, the literature today distinguishes between explicit and implicit attitudes.

Explicit attitudes are usually under conscious control, and they are reported by the person holding them usually *via* self-reports or interviews (Steffens, 2005; Eagly and Chaiken, 2007). In contrast, implicit attitudes are assumed to be automatically activated, sometimes outside of the person’s awareness (Greenwald et al., 2003; Ajzen et al., 2018). One of the most common ways to assess implicit attitudes is the Implicit Association Test (IAT) (Greenwald et al., 1998). The test assesses the strength of automatic associations between concepts (i.e., homosexual people or heterosexual people) and attributes (i.e., good or bad); (Sabin et al., 2015). The IAT’s rationale is that, in general, people will react faster if they perceive the categories as related to one another (congruent condition). Conversely, if they perceive the categories as unrelated to one another (incongruent condition) the reaction time would be slower (Steffens, 2005; Sabin et al., 2009, 2015). One of the strongest advantages of the IAT is that it may enable revealing attitudes and other automatic associations even for subjects who prefer not to expose their attitudes or are unaware of holding them (Greenwald et al., 1998).

Sabin et al. (2015) examined implicit attitudes toward gay men and lesbian women in a variety of health care providers *via* the IAT. Among the providers examined, mental health providers held the weakest negative implicit bias toward gay men and lesbian women while nurses held the strongest. Yet, the researchers claim that it is still unclear whether these differences can be explained by the type of profession, the level of academic training, ongoing work experiences, or the type of applicants accepted to psychology and nursing studies. So far, in our research literature review, we were not able to find studies regarding the differences between psychology and nursing implicit attitudes toward gay men and lesbian women.

There is sufficient empirical evidence to support the conclusion that the nature of the patient-caregiver relationship is one of the most important factors that affect the quality and outcomes of medical and paramedical treatments (Van Boekel et al., 2013). It is also well established that this relationship is influenced by the caregiver’s attitudes toward the patient. However, there is inconsistency regarding the correlation between implicit attitudes and health care providers’ behavior. Several studies found such correlations and suggested further that negative implicit attitudes held by the health care provider may adversely affect essential communication between the caregiver and the patient possibly leading to avoidance of treatment and impaired health, (Penner et al., 2010, 2016; Sabin and Greenwald, 2012; Fitzgerald and Hurst, 2017). In contrast, other studies found no correlation between implicit attitudes and behavior (Oswald et al., 2013; Machery, 2022). This inconsistency is yet unresolved. Therefore, it is important to further investigate the effects of both implicit and explicit attitudes on caregiver behavior.

The primary purpose of the current study was to examine and compare the extent of explicit and implicit attitudes toward gay men and lesbian women among heterosexual undergraduate and graduate, nursing, and psychology students. These are students from health care professions that specialize in physical and mental care. Nursing and psychology were chosen as representatives of physical care (e.g., physicians, nurses), and mental health care (psychologists, social workers) professions. While both professions emphasize a combination of mental and physical support for the patient, they do so in different ways. Nurses are trained to focus mainly on the physical aspects of care (e.g., medication management and administration, patient education, physical examination) while psychologists specialize in psychotherapy for various mental states and conditions that are often related to physical conditions (e.g., anxiety, depression, post-traumatic stress). Additionally, we aimed to assess the contribution of demographic variables such as ethnic origin, religiosity level, gender, age, and extent of familiarity with gay men and lesbian women to these attitudes.

## Materials and method

### Study design

The current study is cross-sectional. It was approved by the Ethics Committee of Ariel University, Israel. It was conducted in a laboratory room at the university. The participants were undergraduate and graduate students from the psychology and nursing departments, recruited on the campus by the researchers.

### Participants and setting

Table 1 displays demographic data collected from all four groups (nursing and psychology, undergraduate and graduate). A total of 140 participants have participated in the study, 35 participants in each group. The study sample included more women than men and the gender gap was larger among nursing students. Most of the participants were born in Israel. The majority of the students (62%) were single. The sample also included a majority of orthodox individuals and the number of participants with secular or traditional orientations was higher among psychology students than among nursing students. Finally, there was a significant difference between the groups in age. Since the assumption of homogeneity of variance was not met for this data, the analysis obtained the *Welch’s* adjusted *F* ratio and the *Games-Howell post hoc* procedure was used. Graduate psychology students were significantly older than undergraduate psychology students (*Mdiff* = –5.29, *p* < 0.001) and significantly older than undergraduate nursing students. In addition, graduate nursing students were significantly older than undergraduate nursing students (*Mdiff* = 5.00, *p* < 0.001) and significantly older than undergraduate psychology students (*Mdiff* = –4.35, *p* < 0.001).

**TABLE 1:** Comparisons of the study groups by demographic characteristics.

		N (Percentage of the total sample)				Statistical test
	
		Undergraduate psychology	Graduate psychology	Undergraduate nursing	Graduate nursing	Value and significance
Variable	Values					
Gender	Men	6 (4.3%)	13 (9.4%)	3 (2.2%)	4 (2.9%)	χ*^2^*(3) = 11.43 *p* = 0.01
	Women	28 (20.1%)	22 (15.8%)	32 (23%)	31 (22.3%)	
Country of birth	Israel	34 (24.5%)	30 (21.6%)	31 (22.3%)	26 (18.7%)	χ*^2^*(3) = 10.24 *p* = 0.02
	other countries	0	5 (3.6%)	4 (2.9%)	9 (6.5%)	
Family status	Single	31 (22.3%)	17 (12.2%)	24 (17.3%)	13 (9.4%)	χ*^2^*(3) = 24.19 *p* < 0.001
	In relationship	3 (2.2%)	18 (12.9%)	11 (7.9%)	21 (15.8%)	
Religious belief	Orthodox	22 (15.8%)	17 (12.2%)	28 (20.1%)	25 (18%)	χ*^2^*(3) = 8.300 *p* = *0.04*
	Non-orthodox	12 (8.6%)	18 (12.9%)	7 (5%)	10 (7.2%)	
Acquaintance with homosexual men or women	Yes	22 (15.8%)	25 (18%)	15 (10.8%)	19 (13.7%)	χ*^2^*(3) = 6.72 *p* = n.s.
	No	12 (8.6%)	10 (7.2%)	20 (14.4%)	16 (11.5%)	
Age	Mean	23.91	29.2	23.26	28.26	*Welch F*(3,70) = 20.481 *p* < *0.001*
	Standard Deviation	1.86	4.5	2.44	6.4	

### Measures

#### Implicit attitudes

Implicit attitudes were assessed using the Implicit Association Test (IAT). In the present study, the participants were timed as they associated symbols and words representing a group (heterosexuality or homosexuality) with words representing either good or bad attributes. It was assumed that heterosexual participants would respond more quickly in the congruent conditions (i.e., symbols and words of heterosexuality with good attributes, and symbols and words of homosexuality with bad attributes) than they would in incongruent conditions (i.e., symbols and words of heterosexuality with bad attributes and symbols and words of homosexuality with good attributes). For the IAT procedure, we used the Banse (2001) protocol. For the good-bad classification, 16 words were used, each representing either positive or negative valence. For the homosexual-heterosexual classification, 3 words (1 for heterosexuality and 2 for homosexuality), along with 6 pictures (2 for heterosexuality and 4 for homosexuality) were used. The stimuli were taken from Harvard University’s Project Implicit Test (Nosek et al., 2007).

The participants were told that they will be asked to sort words and pictures into categories. The experimenter emphasized that they must do the sorting as quickly as possible (if it took participants more than 2000 ms to choose a category, a message appeared, asking them to answer faster in the next trials), and with few mistakes as possible (in case of a mistake in categorization, a red “x” appeared until the right choice was made). At each sequence, stimuli appeared at the center of the screen and participants had to classify them into one of two groups that appeared on the upper left or right side of the screen. The sorting was made by pressing the “E” (left side) or the “I” (right side) keys on the keyboard.

The IAT included two versions with five phases in each. In IAT1, participants were asked to associate bad attributes to the left side of the screen and good attributes to the right side of the screen. In IAT2, participants were asked to associate good attributes to the left side of the screen and bad attributes to the right side of the screen. The participants were asked to do both versions in a randomized order. In each version, the third and fifth blocks were critical (with the exception of the first 20 trails, which were used for training) while the rest were training blocks. The third block of IAT1 and the fifth block of IAT2 were both “homosexual/bad” blocks except for the screen side. The fifth block of IAT1 and the third block of IAT2 were both “homosexual/good” blocks except for the screen side. Table 2 presents the IAT protocol that was used for the current study.

**TABLE 2:** Protocol of the IAT versions and answer keys assignment.

Version	Block	N of trials	Answer key assignment	
			Left key (“E”)	Right key (“I”)
IAT1	1	20	Homosexual	Heterosexual
	2	32	Bad	Good
	**3**	**20 + 52**	**Homosexual/Bad**	**Heterosexual/Good**
	4	20	Heterosexual	Homosexual
	**5**	**20 + 52**	**Heterosexual/Bad**	**Homosexual/Good**
IAT2	1	20	Homosexual	Heterosexual
	2	32	Good	Bad
	**3**	**20 + 52**	**Homosexual/Good**	**Heterosexual/Bad**
	4	20	Heterosexual	Homosexual
	**5**	**20 + 52**	**Heterosexual/Good**	**Homosexual/Bad**

The critical blocks are bolded.

##### Scoring the implicit association test

The third block of IAT1 and the fifth block of IAT2 were aggregated as “homosexual/bad” block and the fifth block of IAT1 and the third block of IAT2 were aggregated as “homosexual/good” blocks. The d-score algorithm (Greenwald et al., 2003) was adjusted for the depended variable. Specifically, for each participant, the reaction time difference between the “homosexual/good” block and the “homosexual/bad” block was computed. Then we divided it by the participant’s standard deviation across the aggregated blocks. The d-score represent implicit attitudes toward gay men and lesbian women. A positive d-score represents negative implicit attitudes, zero represents neutral implicit attitudes and a negative d-score represents positive implicit attitudes. Error rate of 30% in any of the critical blocks was chosen as an unusually high rate of errors and was it was determined as an excluding criterion. Latencies of less than 400 ms or greater than 10,000 ms were recorded as missing. Responses for both correct and incorrect answers were used for the analyses.

#### Explicit attitudes

Explicit attitudes were measured using the Hebrew version of Herek’s Attitudes Toward Lesbians and Gay questionnaire (ATLG; Herek, 1988). The questionnaire included 20 statements regarding gay men and lesbian women. Participants were asked to indicate their agreement on a scale of 1 (“totally disagree”) to 5 (“totally agree”). The questionnaire included ten statements regarding homosexual men (e.g., *“Homosexual men should not be allowed to teach at schools”*) and ten statements regarding homosexual women (e.g., *“Lesbians could not fit into our society”*). For the current study, the sum of the total items in the questionnaire was aggregated. The higher the score, the more negative the explicit attitudes are.

#### Demographic variables

Age, gender, country of birth (dichotomized as Israel or other countries), marital status (dichotomized as single or in a relationship), level of religiosity (dichotomized as orthodox or non-orthodox), and familiarity with homosexual men or women (dichotomized as yes or no) were assessed. Participants’ sexual orientation was measured *via* the Kinsey scale (Kinsey et al., 1948; Kinsey, 1953). Participants were asked to rate their sexual orientation *via* a scale ranging from 0 (*“exclusively heterosexual”*) to 6 (*“exclusively homosexual*”). Participants who scored “2” (*“Predominantly heterosexual, but more than incidentally homosexual”*) or higher, would be excluded from the study. However, none of the subjects scored above “1.”

#### Procedure

The participants were asked to participate in a study aiming to measure attitudes toward gay men and lesbian women. After signing the informed consent form, participants were told that the experiment will include a computerized sorting task and a questionnaire, starting with the computerized IAT task. The IAT test was run in a laboratory room using *Direct-RT* software. They were then asked to complete demographic detail items and the explicit attitudes questionnaires. Data were analyzed using *SPSS v26*.

## Results

### Implicit attitudes

#### Reliability

Internal consistency was measured *via* Cronbach’s alpha. The α coefficients for 25 stimuli were satisfactory for the first and second versions (*Cronbach’s*α ≥ 0.92). Inter-method reliability was measured *via* the correlation between the third block of version one and the fifth block of version two and the correlation between the fifth block of version one and the third block of version two. The correlation between the third block of version one and the fifth block of version two was *r* = 0.62 (*p* < 0.001). The correlation between the fifth block of version one and the third block of version two was *r* = 0.65 (*p* < 0.001). The moderate correlations suggest that although the versions are correlated with each other, they may have a unique variance that needs to be further assessed and was not examined in the current study.

#### Demographic variables

None of the demographic variables had a significant contribution to implicit attitudes.

#### Main results

Two analyses were conducted. First, a one-sample *t*-test to determine whether the d-score across the entire sample was greater than 0. We hypothesized that the sample’s d-score’s mean would be greater than 0, which suggests a negative implicit attitudes toward gay men and lesbian women. Then, we run a 2-way between-subject Analysis of Variance (ANOVA) model (2x2) with profession (Psychology or Nursing) and the level of training (Undergraduate or Graduate) as the independent variables. One participant, from the undergraduate psychology group, was dropped from the analysis due to more than 30% of wrong answers trails in the critical blocks. Thus, the final sample was *n* = *139*. Table 3 shows the results of the statistical analyses.

**TABLE 3:** A 2-way *ANOVA* for the profession, the level of training, and the profession by the level of training interaction on implicit attitudes (*d-score*).

	Profession		Level of training				
	Psychology	Nursing	Undergraduate	Graduate	Profession	Level of training	Profession*Level
	**M** **(SD)**	**M** **(SD)**	**M** **(SD)**	**M** **(SD)**	**F(1,135)** **(η_*p*_^2^)**	**F(1,135)** **(η_*p*_^2^)**	**F(1,135)** **(η_*p*_^2^)**
Total	0.21 (0.83)	0.25 (0.73)	0.11 (0.76)	0.34 (0.78)			
Undergraduate	0.2 (0.74)	0.03 (0.78)			0.09 (0.00)	2.98 (0.02)	2.50 (0.02)
Graduate	0.22 (0.91)	0.47 (0.62)					

First, a one-sample *t-*test yielded a significant effect. The study sample’s d-score mean was greater than 0 (*M* = 0.23, *SD* = 0.78), meaning the study sample had a slower reaction time associating gay men and lesbian women stimuli with good attributes compared with bad attributes [*t*(198) = 3.47, *p* < 0.001, *Cohen’s d* = 0.29]. The *ANOVA* analysis yielded no significant main nor interaction effect. There was no difference in d-score between psychology and nursing students, nor between undergraduate and graduate students.

#### Explicit attitudes

#### Reliability

The ATLG questionnaire included ten statements regarding homosexual men (*Cronbach’s*α = 0.85) and 10 statements regarding homosexual women (*Cronbach’s*α = 0.92). In the current study, we assessed the explicit attitudes toward gay men and lesbian women combined. Thus, we summarized the results of the questionnaire (*Cronbach’s*α = 0.94).

#### Demographic variables

Level of religiosity was found to have a significant contribution to explicit attitudes. Since the normality of errors assumption was violated (right-skewed distribution of the ATLG questionnaire), *Mann-Whitney U* test was adjusted. Orthodox participants (*Med* = 45) had more negative explicit attitudes toward gay men and lesbian women than non-orthodox participants (*Med* = 37); [*Mann-Whitney U* = 1240.50, n*_*Orthodox*_* = 91, n*_*Non*_ _*orthodox*_* = 48, *p* < 0.001].

#### Main results

A 2-way between-subjects Analysis of Covariance (ANCOVA) model (2X2), with the level of religiosity as a covariate, was conducted. Since the normality of errors assumption was violated, explicit attitudes were *log*-transformed before the analysis. The presentation of the groups’ means, and standard deviations are presented in raw scores for better comparisons with the research field. Table 4 shows the results of the statistical analyses.

**TABLE 4:** A 2-way *ANCOVA* (with level of religiosity as a covariate) for the profession, the level of training, and the profession by the level of training interaction on explicit attitudes.

	Profession		Level of training				
	Psychology	Nursing	Undergraduate	Graduate	Profession	Level of training	Profession*Level
	**M** **(SD)**	**M** **(SD)**	**M** **(SD)**	**M** **(SD)**	**F(1,134)** **(ηp2)**	**F(1,134)** **(ηp2)**	**F(1,134)** **(ηp2)**
Total	41.25 (9.63)	48.47 (14.37)	47.14 (14.17)	42.66 (10.77)			
Undergraduate	41.47 (11.41)	52.66 (14.55)			6.80** (0.05)	2.39 (0.02)	5.03* (0.04)
Graduate	41.03 (7.67)	44.29 (13.09)					

*p < 0.05. **p < 0.01.

While the analysis yielded a significant Profession effect, the analysis also yielded a crossover (dis-ordinal) interaction effect. *Post hoc* comparisons, using the *Benjamini-Hochberg* correction, showed that among undergraduate students, nursing students reported holding more negative explicit attitudes toward gay men and lesbian women than psychology students (*p* < 0.001). In addition, among nursing students, undergraduates reported holding more negative explicit attitudes toward gay men and lesbian women than graduates (*p* = 0.01). There were no other significant differences between the study groups.

### Implicit and explicit attitudes correlation

**FIGURE 1:**
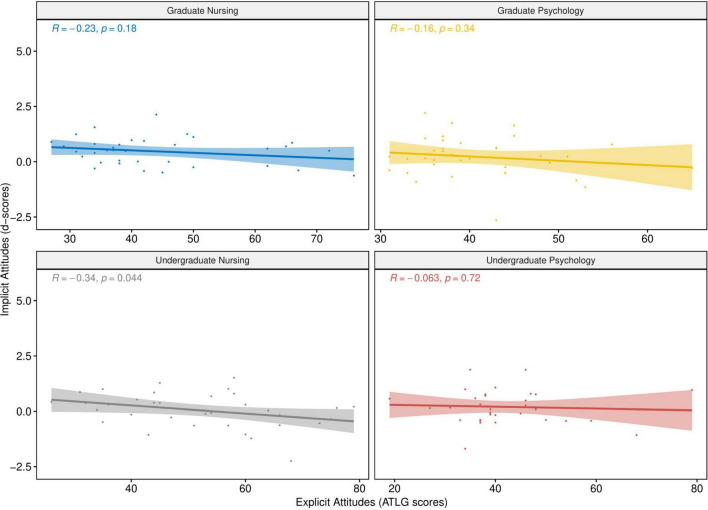
*Pearson* correlation (with a 95% *confidence intervals*) between implicit and explicit attitudes among each of the four study groups.

The correlation between implicit and explicit attitudes was examined within each of the four study groups. As shown in Figure 1, the analyses yielded a significant correlation only among undergraduate nursing students. A weak and negative correlation was found in this group between explicit attitudes scores and the d-scores.

## Discussion

First, all groups, regardless of profession or level of training, held negative implicit attitudes toward gay men and lesbian women. All study groups had more difficulty associating homosexual stimuli with good attributes compared with bad attributes. Second, there were no differences in implicit attitudes between undergraduate and graduate students, nor between psychology and nursing students. Compared with this uniformity, different results were found in explicit attitudes. Among nursing students, undergraduates reported holding more negative explicit attitudes toward gay men and lesbian women than graduates. In addition, among undergraduates, nursing students reported holding more negative explicit attitudes toward gay men and lesbian women than psychology students. Finally, A weak and negative correlation was found among undergraduate nursing students. No correlations were found in any of the other study groups.

While the differences in explicit attitudes associated with the profession add to the existing research literature regarding differences between psychology and nursing students’ attitudes toward minority groups, the results may suggest that psychology and nursing students, at both undergraduate and graduate levels, hold similar negative implicit attitudes toward gay men and lesbian women. A review of the research literature did not elicit any studies comparing psychology and nursing students’ implicit attitudes toward any population with the exception of the study carried out by Waller et al. (2012). The researchers assessed implicit attitudes toward overweight individuals among undergraduate nursing and psychology students. Their analysis yielded no differences in attitudes between the two groups. However, the current findings are in contrast to the results obtained by Sabin et al. (2015) who found that nurses held more negative implicit attitudes toward gay men and lesbian women than mental health care providers. This discrepancy may reflect both cultural differences in attitudes between the two studies as well as the fact that the nurses and mental health care providers included in Sabin’s study were older and more experienced professionals than our student sample.

With regard to explicit attitudes, in the two existing studies, nursing students reported the strongest explicit negative bias toward minority groups. Harling (2017) examined and compared explicit attitudes of nursing students, clinical psychology trainees, social work students, midwifery students, and health and social care students toward illicit drug users. They found that nursing students reported the strongest explicit negative bias toward illicit drug users while clinical psychology students held the weakest. Papadaki et al. (2015) investigated explicit attitudes toward gay men and lesbian women among undergraduate social work, psychology, medical and nursing students. They found that psychology students reported holding more positive explicit negative attitudes toward homosexuals in comparison to nursing students.

While in the present study there was no difference between undergraduate and graduate psychology students’ explicit attitudes, our results suggest an improvement in graduate nursing students’ explicit attitudes, compare with undergraduate nursing students. Studies suggest that knowledge regarding LGBTQ+ health care issues and experience with such patients may lead to an improvement in attitudes toward them (Bowers et al., 2015; Phelan et al., 2017). Cornelius and Carrick (2015) examined undergraduate, graduate, and RN-BSN nursing students’ knowledge and willingness to provide care and comfort to LGBTQ+ patients. Their results suggest that the higher the level of training, the more they will be willing to provide such care. In ^***^, nursing students usually start their graduate degrees after acquiring several years of clinical practice, including three years as part of their undergraduate studies. In contrast, psychology students begin their clinical practice only at their graduates’ studies, as part of their curriculum, following a part-time, four-year internship. Thus, nursing students may have better chances of coming across gay and lesbian patients and may possess more knowledge and experience regarding their health needs. However, this assumption must be tested in future research.

### Conclusion and implications

Medical and paramedical education systems are encouraged to educate students to provide equitable quality care to the entire population. The findings from the current study regarding negative implicit and explicit attitudes toward gay men and lesbian women among future nurses and psychologists refute this view. Thus, we recommend that in order to tackle and reduce the extent and severity of biases and stigmas that exist among medical teams is to intervene from the beginning of medical and nursing education. Medical, nursing and psychology curricula should prepare the future health care providers to provide gay men and lesbian women equitable quality care. This in return, may eventually reduce health disparities among LGBTQ+ patients.

### Limitations

The current study has serval limitations. First, the number of years of clinical practice was not assessed. It is therefore impossible to examine the differences between psychology and nursing students in the extent of their clinical experience, which might have explained some of the findings. Also, we did not assess the students’ knowledge regarding health needs of gay men and lesbian women nor knowledge regarding health disparities among LGBTQ+ population. Finally, the current study did not assess the participants’ adherence to traditional masculinity and femininity, which has been found as a major predictor of implicit attitudes (Salvati et al., 2021).

With regards of the IAT, although it is widely used as an experimental measurement of implicit attitudes, it does possess several limitations (Azar, 2008). First, its test-retest reliability, as in the current study, is not satisfactory. Another limitation is the inconsistency in the literature regarding the correlation between the IAT and medical decision-making (Oswald et al., 2013; Machery, 2022). In addition, the IAT may not reveal attitudes rather than preference to the socially dominant group, even among socially discriminated groups (Arkes and Tetlock, 2004; Blanton and Jaccard, 2008).

## Data availability statement

The datasets presented in this study can be found in online repositories. The names of the repository/repositories and accession number(s) can be found in the article/supplementary material.

## Ethics statement

The studies involving human participants were reviewed and approved by Ariel University. The patients/participants provided their written informed consent to participate in this study.

## Author contributions

OH wrote the manuscript, organized the database, performed the statistical analyses, ran the study, and programed the IAT. YM contributed to the design of the study. AB helped recruit nursing students. TK wrote the first draft of the manuscript and conceptualized the study. All authors contributed to manuscript revision, read, and approved the submitted version.

## Conflict of interest

The authors declare that the research was conducted in the absence of any commercial or financial relationships that could be construed as a potential conflict of interest.

## Publisher’s note

All claims expressed in this article are solely those of the authors and do not necessarily represent those of their affiliated organizations, or those of the publisher, the editors and the reviewers. Any product that may be evaluated in this article, or claim that may be made by its manufacturer, is not guaranteed or endorsed by the publisher.
